# Supplementary material for: Content-rich biological network constructed by mining PubMed abstracts
Source: BMC Bioinformatics. 2004 Oct 8;5:147. doi: 10.1186/1471-2105-5-147 (PMC528731; doi:10.1186/1471-2105-5-147)
Supplement: Additional File 2 — The original results of the above study (non-essential files are deleted to keep the file size under the limit set by BMC bioinformatics). [file 1471-2105-5-147-S2.bz2 › chilibotAdditionalFile2/dip40/136ID8872303E489/html/GAP43_CALM1.html]

 


 **GAP43** and **CALM1** 
  
Found 143 abstracts in PubMed, retrieved 40.  
 

 What does Google say? 
 PDF only 
| .edu only 

---

**Interactive relationship** (e.g. stimulation, inhibition, etc)

**Neutral relationship**- The capacitance of the system is regulated by phosphorylation by protein kinase C, which abrogates interactions between calmodulin  [ **CALM1** ]  and RC3 or GAP 43  [ **GAP43** ] .  Ref: 9396008 Mol Neurobiol, 1997
- This region is not required for activation of isolated Go but is necessary for GAP 43  [ **GAP43** ]  binding to isolated calmodulin  [ **CALM1** ]  and to isolated protein kinase C.  Ref: 9489717 J Neurochem, 1998
- GAP 43  [ **GAP43** ]  also interacted with synaptotagmin and calmodulin  [ **CALM1** ] .  Ref: 9230128 Biochem J, 1997
- Over 60% of the plasma membrane associated calmodulin  [ **CALM1** ]  co localized with B 50  [ **GAP43** ]  GAP 43  [ **GAP43** ]  in a non random distribution.  Ref: 9219979 Neuroscience, 1997
- Calmodulin  [ **CALM1** ]  and de phosphorylated B 50  [ **GAP43** ]  growth associated protein 43  [ **GAP43** ]  GAP 43  [ **GAP43** ]  have been shown to bind in vitro in a molecular complex, but evidence for an in situ association in the nervous system does not exist.  Ref: 9219979 Neuroscience, 1997
- Phosphorylation of GAP 43  [ **GAP43** ]  caused a disruption of its complex with calmodulin  [ **CALM1** ] .  Ref: 10051751 Eur J Neurosci, 1999
- In contrast, unphosphorylated GAP 43  [ **GAP43** ] , which binds calmodulin  [ **CALM1** ] , is always found in parts of the growth cone that are retracting.  Ref: 9133376 J Neurosci, 1997
- The cleavage occurs in the region responsible for the interaction of GAP 43  [ **GAP43** ]  with calmodulin  [ **CALM1** ] .  Ref: 11092958 Biochemistry Mosc , 2000
- The physiological significance could be that this association regulates the local availability of both B 50  [ **GAP43** ]  GAP 43  [ **GAP43** ]  and calmodulin  [ **CALM1** ]  for other interactions.  Ref: 9219979 Neuroscience, 1997
- Apo calmodulin  [ **CALM1** ]  binds typical target sequences from skeletal muscle myosin light chain kinase and neuromodulin  [ **GAP43** ]  with Kd approximately 70 90 nM at low ionic strength.  Ref: 11206069 Protein Sci, 2000
- NAP 22, a neuronal tissue enriched acidic membrane protein, is a Ca 2 dependent calmodulin  [ **CALM1** ]  binding protein and has similar biochemical characteristics to GAP 43  [ **GAP43** ]  neuromodulin  [ **GAP43** ] .  Ref: 10958980 Neurosci Res, 2000
- GAP 43  [ **GAP43** ]  is bound by calmodulin  [ **CALM1** ]  when Ca2 levels are low, and releases the calmodulin  [ **CALM1** ]  when Ca2 levels rise, suggesting that calmodulin  [ **CALM1** ]  may act as a negative regulator of GAP 43  [ **GAP43** ]  during periods of low activity in the neurons.  Ref: 9742146 J Neurosci, 1998
- Previously, sequence analysis of Sp17 revealed that Sp17 amino acids 108 137 were 52% identical to the calmodulin  [ **CALM1** ]  binding domain of neuromodulin  [ **GAP43** ]  and contained an IQ motif found in other calmodulin  [ **CALM1** ]  binding proteins.  Ref: 9986726 Dev Biol, 1999
- A growing family of proteins is regulated by protein kinase C and calmodulin  [ **CALM1** ]  through IQ domains, a regulatory motif originally identified in neuromodulin  [ **GAP43** ]  Alexander, K.  Ref: 9341188 J Biol Chem, 1997
- Analogously to the nervous system, phosphoprotein B 50  [ **GAP43** ]  may serve a local function involving transmembrane signalling by means of calmodulin  [ **CALM1** ]  binding of phosphoprotein B 50  [ **GAP43** ]  and or phosphoprotein B 50  [ **GAP43** ]  phosphorylation and dephosphorylation.  Ref: 9618702 Neurol Res, 1998
- GAP 43  [ **GAP43** ]  is an abundant intracellular growth cone protein that can serve as a PKC substrate and regulate calmodulin  [ **CALM1** ]  availability.  Ref: 10662636 Development, 2000
- The protein kinase C PKC substrate GAP 43  [ **GAP43** ]  is already expressed in neural precursor cells, colocalizes with PKCeta and binds calmodulin  [ **CALM1** ] .  Ref: 10051751 Eur J Neurosci, 1999
- Double labeling using nonradioactive ISH and immunofluorescence revealed that both GAP 43  [ **GAP43** ]  and SCG10 mRNAs were coexpressed with the alpha subunit of type II calcium calmodulin  [ **CALM1** ]  dependent protein kinase, indicating that both mRNAs are expressed also in koniocellular neurons in the LGN.  Ref: 10934252 J Neurosci, 2000
- Thus, GAP 43  [ **GAP43** ]  regulates an oocyte signaling cascade via coordinated, simultaneous G protein activation and interaction with either calmodulin  [ **CALM1** ]  or protein kinase C.  Ref: 9489717 J Neurochem, 1998
- Since normally calmodulin  [ **CALM1** ]  is largely present in the cytoplasm, these data suggest that calmodulin  [ **CALM1** ]  has been concentrated at the plasma membrane of unmyelinated axons, most probably by B 50  [ **GAP43** ]  GAP 43  [ **GAP43** ] .  Ref: 9219979 Neuroscience, 1997
- Neuromodulin  [ **GAP43** ]  GAP 43  [ **GAP43** ] , neurogranin RC3, and PEP 19 are small acid stable proteins that bind calcium poor calmodulin  [ **CALM1** ]  through a loosely conserved IQ motif.  Ref: 11414283 Mol Neurobiol< MedlineTA>Mol Neurobiol, 1997
- Instead, GAP 43  [ **GAP43** ]  appears to mediate RGC axon interaction with guidance cues in the lateral diencephalic wall, suggesting possible involvement of PKC and calmodulin  [ **CALM1** ]  signaling during optic tract formation.  Ref: 10662636 Development, 2000
- This mutation blocks GAP 43  [ **GAP43** ]  binding to both protein kinase C and calmodulin  [ **CALM1** ] .  Ref: 9489717 J Neurochem, 1998
- Calmodulin  [ **CALM1** ] , which binds to GAP 43  [ **GAP43** ]  and inhibits its phosphorylation by protein kinase C, abolished the effect of TPA.  Ref: 9619293 Cell Mol Neurobiol, 1998
- Administration of palmitoylcarnitine, although did not change the subcellular distribution of GAP 43  [ **GAP43** ] , decreased its phosphorylation, which could regulate other signal transduction pathways calmodulin  [ **CALM1** ]  and G 0 dependent.  Ref: 12061781 Biochem Biophys Res Commun, 2002
- We propose a model wherein RC3 and GAP 43  [ **GAP43** ]  regulate calmodulin  [ **CALM1** ]  availability in dendritic spines and axons, respectively, and calmodulin  [ **CALM1** ]  regulates their ability to amplify the mobilization of Ca2 in response to metabotropic glutamate receptor stimulation.  Ref: 9396008 Mol Neurobiol, 1997

**Non-interactive relationship** (e.g. studied together, co-existance, homology, etc.)

- Nonisotopic in situ hybridization techniques were used to define the subcellular localization of representative mRNAs including beta tubulin, low molecular weight neurofilament protein, high molecular weight microtubule associated protein, growth associated protein 43  [ **GAP43** ] , the alpha subunit of calcium calmodulin  [ **CALM1** ]  dependent protein kinase II, and poly mRNA.  Ref: 9322162 J Neurobiol, 1997
- Since the in situ phosphorylation of the calmodulin  [ **CALM1** ]  binding proteins GAP 43  [ **GAP43** ]  B 50  [ **GAP43** ]  and RC3 changes during both LTD and long term potentiation, these proteins may form part of the link between the Ca 2 signal and Ca 2 calmodulin  [ **CALM1** ]  dependent processes implicated in long term potentiation and LTD.  Ref: 10867003 J Biol Chem, 2000
- The proteolysis disturbs some properties characteristic for whole GAP 43  [ **GAP43** ]  molecules, in particular, calmodulin  [ **CALM1** ]  binding and Ser 41 phosphorylation, when the cleavage occurs at 41st residue.  Ref: 11274743 Neurosci Res, 2001
- To better understand the molecular basis of this neuroanatomical alteration, the present study examined three developmentally regulated protein kinase C substrate mRNAs that are highly expressed in hippocampal granule cells during mossy fiber outgrowth GAP 43  [ **GAP43** ] , a presynaptic substrate implicated in axonal outgrowth, RC3 neurogranin, a postsynaptic substrate implicated in calmodulin  [ **CALM1** ]  signaling, and MARCKS like protein MLP, which binds calmodulin  [ **CALM1** ]  and filamentous actin in neurons and glial cells.  Ref: 12697986 Dev Neurosci, 2002
- Immunofluorescence showed that seven days post sciatic nerve crush the calmodulin  [ **CALM1** ]  immunoreactivity, similar to B 50  [ **GAP43** ]  GAP 43  [ **GAP43** ]  immunoreactivity, was intense in unmyelinated axon shafts located proximal to the site of injury of the regenerating nerve.  Ref: 9219979 Neuroscience, 1997
- Because brain derived raft contains two calmodulin  [ **CALM1** ]  binding proteins, GAP 43  [ **GAP43** ]  and NAP 22 as the major protein components, the raft domain is assumed to be important in the Ca 2 signaling.  Ref: 11319767 J Neurosci Res, 2001
- The Ca 2 rises are thought to induce the activation of  **GAP43**  and Ca 2 calmodulin  [ **CALM1** ]  dependent kinase II, molecules implicated in the modulation of cytoskeletal remodelling.  Ref: 10964748 Mol Cell Biol Res Commun, 2000
- Peptides derived from the membrane and calmodulin  [ **CALM1** ]  binding domains of the myristoylated alanine rich C kinase substrate and neuromodulin  [ **GAP43** ]  were previously found to be in extended conformations.  Ref: 9521661 Biochemistry, 1998
- To explore a putative function of B 50  [ **GAP43** ]  GAP 43  [ **GAP43** ] , namely, the capacity of binding calmodulin  [ **CALM1** ]  to the plasma membrane, we examined the ultrastructural distribution of calmodulin  [ **CALM1** ]  in the proximal unmyelinated axon shafts of this model, using double immunolabelling and detection by fluorescent or gold probes conjugated to second antibodies.  Ref: 9219979 Neuroscience, 1997
- To identify the function of GAP 43  [ **GAP43** ]  during activity dependent increases in Ca2 levels, when it is not bound to calmodulin  [ **CALM1** ] , we sought proteins with which GAP 43  [ **GAP43** ]  interacts in the presence of Ca2.  Ref: 9742146 J Neurosci, 1998
- This resemblance and the fact that GAP 43  [ **GAP43** ]  also has been proposed to regulate calmodulin  [ **CALM1** ]  availability raise the possibility that calmodulin  [ **CALM1** ]  function is involved in CNS midline axon guidance in both vertebrates and invertebrates.  Ref: 9852588 J Neurosci, 1998
- Ultrastructural co localization of calmodulin  [ **CALM1** ]  and B 50  [ **GAP43** ]  growth associated protein 43  [ **GAP43** ]  at the plasma membrane of proximal unmyelinated axon shafts studied in the model of the regenerating rat sciatic nerve.  Ref: 9219979 Neuroscience, 1997
- Small proteins that modulate calmodulin  [ **CALM1** ]  dependent signal transduction effects of PEP 19, neuromodulin  [ **GAP43** ] , and neurogranin on enzyme activation and cellular homeostasis.  Ref: 11414283 Mol Neurobiol< MedlineTA>Mol Neurobiol, 1997
- Although neuromodulin  [ **GAP43** ] , neurogranin, and PEP 19 were early representatives of the small IQ motif containing protein family, newer examples have come to light that expand the number of cellular systems through which the IQ peptide calmodulin  [ **CALM1** ]  interaction could regulate biological processes including gene transcription.  Ref: 11414283 Mol Neurobiol< MedlineTA>Mol Neurobiol, 1997
